# Epigenetic differences in the tumor suppressor genes *MLH1* and *p16INK4a* between Nepalese and Swedish patients with colorectal cancer

**DOI:** 10.1515/iss-2023-0039

**Published:** 2024-07-03

**Authors:** Bikal Ghimire, Göran Kurlberg, Peter Falk, Yogendra Singh, Yvonne Wettergren

**Affiliations:** Department of GI and General Surgery, Maharajgung Medical Campus, Institute of Medicine, Tribhuvan University, Kathmandu, Nepal; Department of Surgery, Institute of Clinical Sciences, Sahlgrenska Academy, University of Gothenburg, Gothenburg, Sweden; Department of Surgery, Region Västra Götaland, Sahlgrenska University Hospital, Gothenburg, Sweden

**Keywords:** colorectal cancer, *MLH1*, *p16INK4a*, methylation, mucosa, CpG sites

## Abstract

**Objectives:**

Colorectal cancer (CRC) is one of the most prevalent cancer types worldwide, exhibiting significant variance in incidence rates across different ethnicities and geographical regions. Notably, there is a rising incidence of CRC among younger adults, particularly evident in advanced stages, with a more pronounced trend observed in developing nations. Epigenetic alterations potentially play a role in the early onset of CRC and could elucidate interpopulation disparities. This study aimed to examine DNA methylation levels in the tumor suppressor genes *MLH1* and *p16INK4a*, comparing Nepalese and Swedish patients with CRC.

**Methods:**

Patients who underwent CRC surgery at Tribhuvan University Teaching Hospital, Nepal (n=39), and Sahlgrenska University Hospital, Sweden (n=39) were included. Demographic and clinicopathological data were analyzed, and pyrosequencing was employed to determine methylation levels in the *MLH1* promoter region and the first exon of *p16INK4a* in tumor tissues and adjacent mucosa located 10 cm from the tumor site. Subsequently, methylation status was compared between Nepalese and Swedish patients and correlated with clinicopathological parameters.

**Results:**

Nepalese and Swedish patients displayed equal levels of *MLH1* and *p16INK4a* methylation in tumors, but Nepalese patients exhibited a significantly higher level of *MLH1* methylation in mucosa compared to Swedish patients (p=0.0008). Moreover, a greater proportion of Nepalese patients showed *MLH1* methylation in mucosa compared to Swedish patients (31 vs. 2.6 %). Aberrant methylation of *p16INK4a* was also observed in the mucosa of Nepalese patients, characterized by high methylation at specific sites rather than uniform methylation across CpG sites. There were no significant differences in methylation levels based on tumor location among Nepalese patients, whereas Swedish patients exhibited higher methylation in right- compared to left-sided colon tumors. Swedish patients showed an increase in *p16INK4a* methylation in tumors with advancing age.

**Conclusions:**

Nepalese and Swedish patients displayed equal levels of *MLH1* and *p16INK4a* methylation in tumors. In contrast, Nepalese patients had a higher level of *MLH1* methylation as well as aberrant methylation of *p16INK4a* in mucosa compared to Swedish patients. These epigenetic differences may be linked to environmental and lifestyle factors. Ongoing research will further explore whether hypermethylation in the mucosa of Nepalese patients is associated with tumorigenesis and its potential utility in screening high-risk patients or predicting recurrence.

## Introduction

Colorectal cancer (CRC) ranks as the third most prevalent cancer disease worldwide, yet its incidence rates exhibit a staggering tenfold variation globally. Notably, European societies report the highest rates, ranging between 40 and 90 cases per 100,000 individuals, while many African regions, like middle Africa, demonstrate significantly lower rates, at approximately 12 cases per 100,000 [1]. Similarly, South Asia presents a diverse landscape, with affluent societies such as South Korea, Japan, and Singapore reporting higher rates compared to lower-income countries in the region [2]. In India, the age-adjusted incidence rate, standardized against a global population standard, drops to as low as 4 cases per 100,000 individuals [2].

The primary risk factor attributed to the onset of CRC is advancing age [3]. European studies estimate a mean age of 72 years among patients with CRC, while in low-income Asian countries, this figure ranges from 43.7 to 51.2 years [4, 5]. Disparities in age between affluent and low-income nations may significantly contribute to the notable divergence in CRC incidence rates within these populations. Interestingly, while there has not been a general increase in CRC incidence over recent decades, there has been a distinct rise observed among individuals under 50 years of age [6]. Additionally, a decline in the age of CRC diagnosis is evident in Asian countries [7]. A retrospective analysis spanning a decade (1999–2008) at a tertiary hospital in Nepal uncovered a noteworthy trend: the mean age of patients with CRC decreased from 34±4.7 years in the initial 5 years to 31±5.1 years in the subsequent half, indicating a declining trend in the age at diagnosis [8].

Numerous studies have delved into the genetic and epigenetic changes underlying the onset of CRC. Of particular interest is DNA hypermethylation, a crucial process implicated in the suppression of tumor suppressor genes. This aberrant methylation of CpG-rich promoter regions of tumor suppressor genes leads to transcriptional inactivation, a hallmark of CRC development [9]. These alterations manifest early and have been discerned in macroscopically normal mucosa adjacent to both benign and malignant tumors. Primarily, they occur at cytosine bases within CpG dinucleotides outside CpG islands [10]. Additionally, DNA replication errors in microsatellite repeat sequences can result in microsatellite instability (MSI), observed in 10–15 % of sporadic CRC cases, signaling deficiencies in DNA mismatch repair (MMR) genes. Epigenetic modifications in the MutL homolog 1 gene (*MLH1*) frequently contribute to MSI, particularly through alterations in its promoter region, which is widely recognized as an early event in CRC tumorigenesis [11], [12], [13].

Frequent hypermethylation has also been documented in the INK4b-ARF-INK4a locus, which encodes two critical cyclin-dependent kinase inhibitors, *p15INK4a* and *p16INK4a*, both pivotal in regulating the G1 phase of the cell cycle. Within this locus, an additional protein, *p14ARF* (alternative reading frame), is also encoded [14]. A prior investigation revealed that hypermethylation of the *p16INK4a* gene promoter was detected in 36 % of mucosa samples obtained 10 cm from the tumor in patients with CRC [15]. Notably, hypermethylation of *p16INK4a* in mucosa correlated with poorer patient outcomes, suggesting that epigenetic changes in the surrounding mucosa might precede genetic alterations in the early stages of carcinogenesis [13].

While epigenetics has been extensively studied in Western populations, the association between epigenetics and CRC remains largely unexplored in resource-constrained countries such as Nepal. This study aimed to bridge this gap by comparing DNA methylation levels in two commonly hypermethylated tumor suppressor genes, *MLH1* and *p16INK4a*, within the tumor and matched mucosa of patients with CRC from both Nepal and Sweden. Additionally, the study aimed to analyze the correlation between the methylation data and the demographic and clinicopathological profiles of the patients.

## Patients and methods

### Patients

Nepalese patients (n=39) who underwent surgery for sporadic (nonhereditary) CRC at Tribhuvan University Teaching Hospital during 2013–2015, and from whom there were accessible tissues and clinical data, were consecutively enrolled without any bias in patient selection. These Nepalese patients were matched for age and sex with 39 Swedish patients who underwent CRC surgery at Sahlgrenska University Hospital. Demographic and clinicopathological data, including age, sex, tumor site, tumor stage, tumor grade, histopathological type, and lymph node status, were recorded. The tumor location was classified as the right colon (cecum, ascending colon, hepatic flexure, and proximal third of the transverse colon), left colon (distal transverse colon, splenic flexure, descending colon, and sigmoid colon), and rectum [16].

### Tissue sampling

Tissue samples were collected during surgery from both tumor sites and macroscopically normal-appearing mucosa situated 10 cm away from the tumor. Tissue samples from Swedish patients were snap-frozen in liquid nitrogen and stored at −80 °C until analysis. In contrast, tissues from Nepalese patients were formalin-fixed and paraffin-embedded (FFPE). To ensure the reliability of the results, methylation levels were assessed and compared in five matched fresh-frozen and FFPE tumor tissues (Supplementary Table 1). Hematoxylin and eosin (H&E) staining was employed for histological confirmation of both FFPE tumor and mucosa tissues.

### DNA extraction

Genomic DNA was isolated from frozen tissue using Qiagen AllPrep DNA/RNA/Protein Kit and from 10 μm tissue sections using the Qiagen FFPE All prep DNA/RNA Kit, according to the manufacturer’s instructions. The DNA samples were kept at −20 °C until analysis.

### DNA denaturation and bisulfite conversion

Two hundred ng of genomic DNA from each sample were used for bisulfite conversion and bisulfite treatment was performed using EpiTecht^®^ Fast Bisulfite Kit (Qiagen, Sollentuna, Sweden) according to the manufacturer’s protocol.

### Measurement of methylation levels

The methylation levels of the *MLH1* and *p16INK4a* genes were quantified using pyrosequencing and PyroMark Q24 assays (Qiagen, Sollentuna, Sweden). The *MLH1* sequence to analyze included five CpG sites: YGGATAGYGATTTTTAAYGYGTAAGYGTATA.

The *p16INK4a* sequence to analyze included six CpG sites: TYGTTAAGTGTTYGGAGTTAATAGTATTTTTTTYGAGTATTYGTTTAYGGYGT; however, only five CpG sites were included in the analyses due to the presence of a single nucleotide polymorphism at the fifth CpG site position. The polymerase chain reactions (PCR) were performed according to the manufacturer’s instructions.

### Preparation of samples for pyrosequencing

One μL of Sepharose beads were mixed with 40 μL of binding buffer and 29 μL of water in an Eppendorf tube. About 60 μL of this mix were added to 20 μL of PCR products in a 96 well plate and agitated at 1,500 rpm for 10 min. The sequencing primers were diluted to 0.375 μM with PyroMark annealing buffer and 20 μL of this solution were then transferred to the PyroMark Advanced Q24 Plate. The washes were performed using the vacuum station according to the manufacturer’s instruction. To anneal the samples to sequencing primers, the temperature was increased to 80 °C for 5 min and the plates were then immediately transferred to the PyroMark Advanced Q24 instrument for processing.

### Pyrosequencing

Pyrosequencing of the purified single stranded PCR products and CpG site quantification was accomplished by the PyroMark Q24 and related software (Qiagen). The CpG sites were investigated in both mucosa and tumor samples. Each CpG site was assigned a percentage of methylation by evaluating the C/T ratio. The mean percentage of methylation across the CpG sites was obtained for each analyzed gene. Representative pyrograms are presented in Supplementary Figures 1 and 2. Hypermethylation was defined as a mean methylation value above the highest value found at any CpG site in normal mucosa obtained from controls in a previous study. This value was 5 % for the *MLH1* gene and 6 % for the *p16INK4a* gene (data not shown).

### Statistical analysis

The obtained data were analyzed by statistical modeling using the commercial software JMP 15.0.0 (SAS Institute, 2019). Data are presented as mean values with SEM bars or as medians and ranges. Differences between groups were assessed using the nonparametric Wilcoxon/Kruskal–Wallis test. p-values <0.05 were considered significant. No correction for multiple testing was done.

## Results

In the study, 56 % of participants were male, and 44 % were female. The median age was 53 years, ranging from 19 to 78 years. Stage III CRC was predominant in the Nepalese group (54 %) compared to the Swedish group (38 %). The majority of the Nepalese patients exhibited well-differentiated tumors (41 %), whereas moderately differentiated tumors were more common among Swedish patients (53 %). Colon cancer represented 74 % of cases among Nepalese patients and 62 % among Swedish patients, with the predominant site of lesions being the right colon (Table 1).

**Table 1:** Patient and tumor characteristics.

	Nepalese patients	Swedish patients
Median age, years (range)	52 (20–78)	53 (19–78)

Sex, n, %		

Male	22 (56.4)	22 (56.4)
Female	17 (43.6)	17 (43.6)

Tumor stage, n, %		

I	3 (8.6)	6 (15.4)
II	13 (37.1)	11 (28.2)
III	19 (54.3)	15 (38.5)
IV	0	7 (17.9)

Tumor differentiation, n, %		

Well	14 (41.2)	2 (5.3)
Moderate	13 (38.2)	20 (52.6)
Low	7 (20.6)	11 (28.9)
Mucinous	0	5 (13.2)

Tumor location, n, %		

Right side of colon	18 (47.4)	17 (43.6)
Left side of colon	10 (26.3)	7 (17.9)
Rectum	10 (26.3)	15 (38.5)

### *MLH1* methylation at individual CpG sites

**Figure 1:**
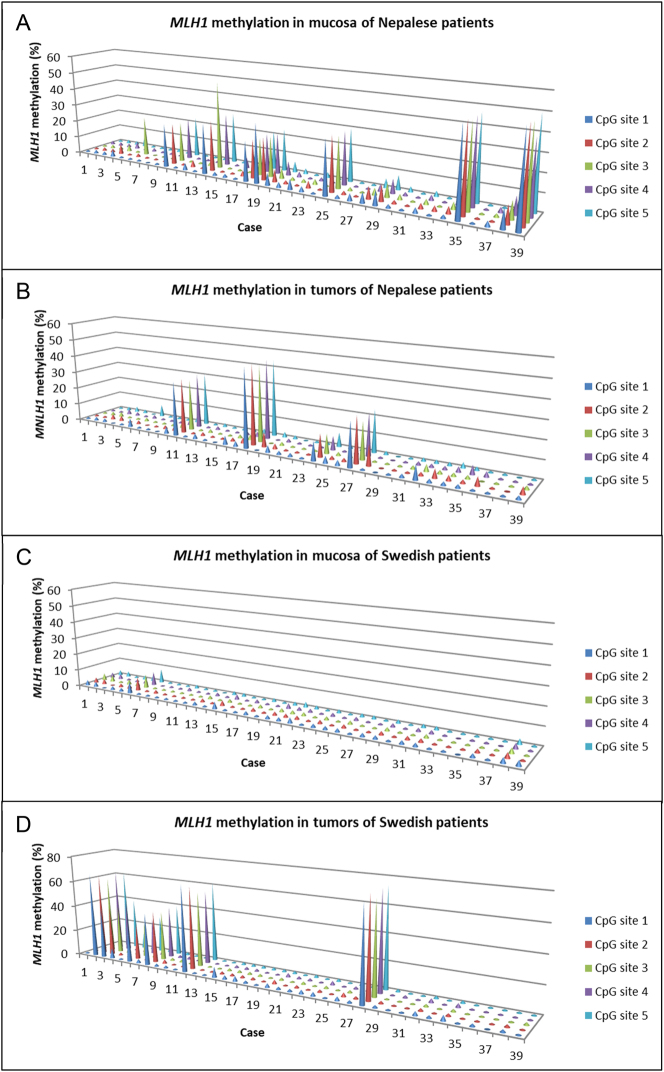
*MLH1* methylation at each CpG site in mucosa and tumor tissue of Nepalese patients (A and B) and Swedish patients (C and D) with colorectal cancer.

*MLH1* methylation levels could be determined in all samples from both the Nepalese and Swedish patient cohorts. Distinct variations in *MLH1* methylation levels between the mucosa and tumors of cohorts were evident at individual CpG sites (Figure 1, Supplementary Table 2). In mucosa samples from Nepalese patients, *MLH1* methylation levels ranged from 0 to 50 %, contrasting with 0 to 8% in Swedish patients. *MLH1* methylation levels in tumor samples ranged from 0 to 46 % and 0 to 74 % in the two patient cohorts, respectively.

### *p16INK4a* methylation at individual CpG sites

**Figure 2:**
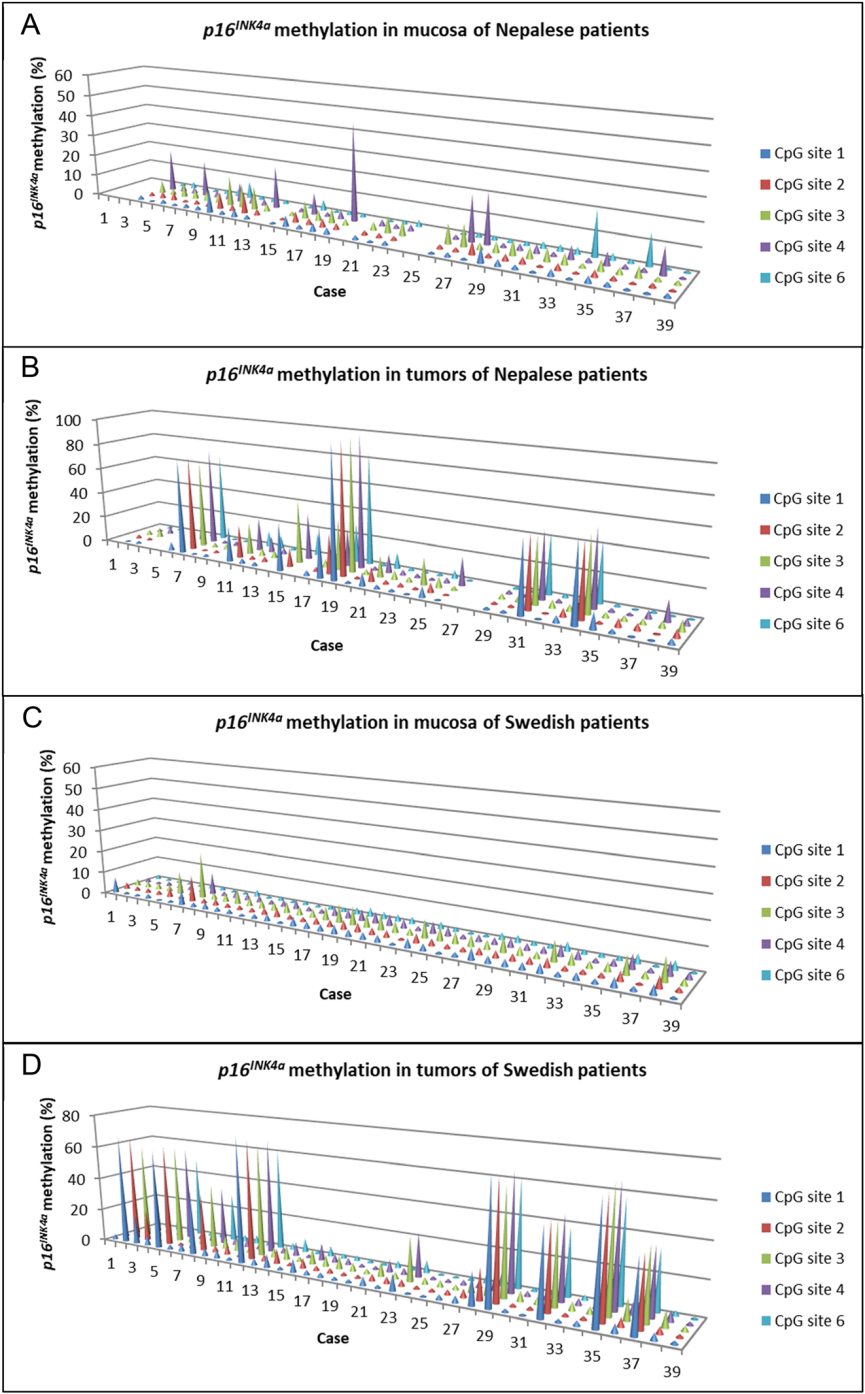
*p16INK4a* methylation at each CpG site in mucosa and tumor tissue of Nepalese patients (A and B) and Swedish patients (C and D) with colorectal cancer. CpG site 5 was not included due to a single nucleotide polymorphism at this position.

**Figure 3:**
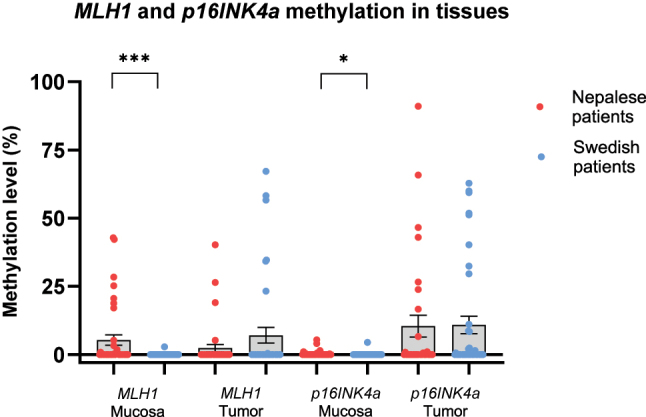
Scatter dot plots showing *MLH1* and *p16INK4a* methylation levels in mucosa and tumor tissues of (A) Nepalese and (B) Swedish patients with colorectal cancer. Data are presented as mean values with SEM bars.

*p16INK4a* methylation was assessable in all samples from Swedish patients and in 32 out of 39 mucosa samples and 31 out of 39 tumor samples from Nepalese patients. In contrast to the generally uniform methylation pattern observed for *MLH1* in mucosa, there was notable variability in methylation levels among different CpG sites for *p16INK4a*, particularly with elevated levels at CpG sites 4 and 6 in mucosa samples from Nepalese patients (Figure 2). Methylation levels of *p16INK4a* ranged from 0 to 46 % in mucosa samples from Nepalese patients, contrasting with 0 to 21 % in Swedish patients. Although *p16INK4a* hypermethylation was more prevalent in tumor samples from Swedish patients, the highest methylation level was detected in a tumor from one of the Nepalese patients (case 19, Figure 3B). Methylation levels of *p16INK4a* in tumors ranged from 1 to 100 % and from 1 to 75 % in the Nepalese and Swedish patient cohorts, respectively.

### *MLH1* and *p16INK4a* methylation in mucosa and tumor tissue

The mean methylation levels of *MLH1* and *p16INK4a* in mucosa and tumors are illustrated in Figure 3. As shown, the methylation levels of *MLH1* and *p16INK4a* were significantly higher in mucosa of Nepalese, compared to Swedish patients. It should be noted though, that the elevated methylation levels of *p16INK4a* in mucosa of Nepalese patients primarily originated from high methylation at CpG site 4, as depicted in Figure 2. Upon excluding this site from the mean value calculations, the difference between the two groups ceased to be statistically significant. There was no significant difference in *MLH1* or *p16INK4a* methylation levels within tumors between the two patient cohorts.

### Methylation according to age

**Figure 4:**
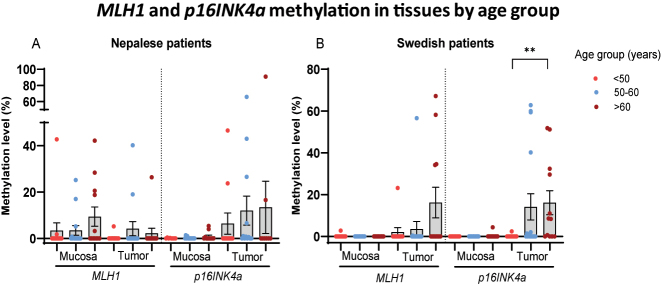
Scatter dot plots showing *MLH1* and *p16INK4a* methylation levels in mucosa and tumor tissues of (A) Nepalese and (B) Swedish patients with colorectal cancer by age group. Data are presented as mean values with SEM bars.

Patients were stratified into three age groups (<50, 50–60, and >60 years old) and assessed for *MLH1* and *p16INK4a* methylation in both mucosa and tumor tissue. The results are presented in Figure 4. There was no significant difference in methylation levels between age groups within the Nepalese patient cohort. However, Swedish patients aged over 60 years exhibited a higher *p16INK4a* methylation level compared to those younger than 50 years (p=0.0046). Furthermore, Nepalese patients had a higher *MLH1* methylation level in mucosa compared to Swedish patients in the age groups of 50–60 (p=0.011) and >60 (p=0.016). The two Swedish patients displaying either *MLH1* or *p16INK4a* methylation in mucosa belonged to the youngest age group. Additional details regarding the percentages of Nepalese and Swedish patients with *MLH1* hypermethylation at respective CpG sites in mucosa and tumor tissue, segmented by age group, can be found in Supplementary Table 3.

**Figure 5:**
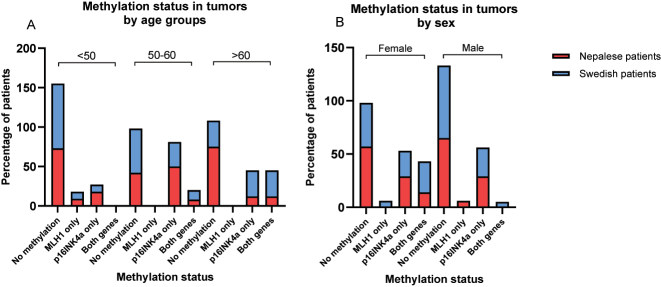
Bars and boxes comparing the *MLH1* and *p16INK4a* methylation status in tumors deriving from Nepalese and Swedish patients with colorectal cancer by (A) age group and (B) sex. The methylation status is classified as follows: no methylation, exclusive *MLH1* methylation, exclusive *p16INK4a* methylation, or methylation in both genes.

Among Swedish patients, the percentage of individuals lacking both *MLH1* and *p16INK4a* methylation in tumor samples decreased with age, dropping from 82 % in the <50 years age group to 33 % in the >60 years age group (Figure 5A). Conversely, in the Nepalese patient cohort, as much as 75 % of patients in the >60 years age group lacked methylation of both genes.

### Methylation according to sex

A notable percentage of Nepalese patients exhibited *MLH1* methylation in mucosa without concurrent methylation in tumor tissue, irrespective of gender, whereas the single Swedish patient (female) with *MLH1* methylation in mucosa also displayed methylation in tumor (Supplementary Figure 3A). Conversely, the proportion of Nepalese patients with methylation exclusively in tumor tissue was low among both females and males (5.9 vs. 9.1 %). In contrast, a higher percentage of Swedish female patients (29.4 %) showed *MLH1* methylation exclusively in tumor tissue compared to males (4.5 %).

Exclusive *p16INK4a* methylation in mucosa without concurrent methylation in tumor tissue was solely detected in female Nepalese patients (Supplementary Figure 3B). However, some patients, both female and male, exhibited methylation in both mucosa and tumor. Similarly, a subset of both female and male patients within the Nepalese cohort displayed exclusive *p16INK4a* methylation within tumor tissue. Conversely, in the Swedish cohort, almost twice as many females as male patients displayed *p16INK4a* methylation exclusively in tumor tissue; however, one male patient exhibited *p16INK4a* methylation in both tumor and mucosa. Upon analyzing the sex distribution based on the presence of both *MLH1* and *p16INK4a* hypermethylation in tumor tissue, it was more prevalent among females than males in both patient cohorts (Figure 5B). The highest frequency was observed among Swedish females (29.4 %).

### Methylation according to tumor location

**Figure 6:**
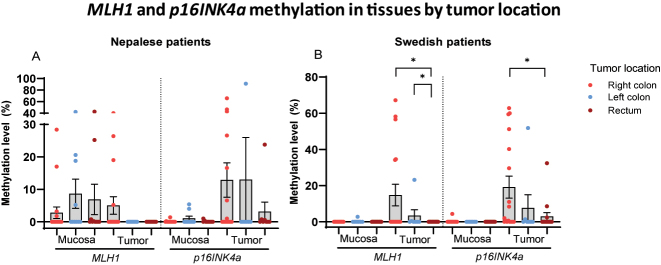
Scatter dot plots showing *MLH1* and *p16INK4a* methylation levels in mucosa and tumor tissues of (A) Nepalese and (B) Swedish patients with colorectal cancer by tumor site. Data are presented as mean values with SEM bars.

No statistically significant difference was observed in the methylation levels of *MLH1* or *p16INK4a* in mucosa or tumor tissues among Nepalese patients based on tumor location (Figure 6A). In Swedish patients, however, significantly higher methylation levels of both genes were detected in tumor samples obtained from the right side of the colon (Figure 6B).

### Methylation according to tumor stage and differentiation

No significant differences in methylation levels were observed across tumor stages, except for *MLH1* methylation in stage III tumors of Swedish patients, which exhibited higher levels compared to other stages (p=0.049). Furthermore, no significant differences in methylation levels were noted based on tumor differentiation.

## Discussion

In this study, we compared the methylation status of the tumor suppressor genes *MLH1* and *p16INK4a* in colorectal tumor tissue and matching mucosa samples from Nepalese and Swedish patients with CRC. Our findings revealed a significantly higher frequency of DNA methylation in the mucosa of Nepalese patients compared to Swedish patients. Additionally, the methylation levels observed in some mucosa samples from Nepalese patients were notably elevated compared to previous reports [17]. The underlying reason for this unexpectedly high methylation level in the mucosa of Nepalese patients remains unclear, but it may be linked to environmental and lifestyle factors known to influence gene regulation through epigenetic mechanisms, including DNA methylation. Such factors encompass age, sex, diet, lifestyle choices, environmental exposures, and medications [18, 19] as discussed below.

Age-related promoter hypermethylation has been documented for several tumor suppressor genes, including the mismatch repair gene *MLH1*, which contributes to 15–30 % of stomach and colon carcinomas [20]. Additionally, it is known that hypermethylation is more prevalent among females than males. Consequently, age and sex differences between patient groups may yield disparate results. However, in this study, the two patient cohorts were matched for these covariates; thus, any observed differences in methylation status between groups would likely be attributed to other factors [18]. Nevertheless, *MLH1* hypermethylation at all five analyzed CpG sites in DNA derived from mucosa was more common in older Nepalese patients. Moreover, the percentage of patients with methylation of both *MLH1* and *p16INK4a* in tumors was observed to increase with age in both Nepalese and Swedish patients. Consistent with previous reports, both *MLH1* and *p16INK4a* methylation in tumor tissue was more prevalent in females compared to males among Swedish patients [21, 22]. *MLH1* methylation in the mucosa of Nepalese patients was also more prevalent in females. However, the methylation status in tumors of Nepalese patients did not follow this pattern, as cases with *MLH1* and *p16INK4a* hypermethylation in tumors were equally distributed among female and male patients.

In addition to age and sex, the occurrence of hypermethylation may also be influenced by tumor location. Typically, hypermethylation is more prevalent in tumors situated on the right side compared to those on the left side of the colon [23]. Consistent with previous findings, the results of this study revealed a higher level of methylation in right-sided colon tumors among both Nepalese and Swedish patients. However, methylation levels in mucosa samples did not exhibit variations based on tumor location.

There was no observed correlation between the methylation levels in mucosa and tumor samples within any of the patient cohorts. This finding aligns with similar results reported by Coppede et al., who analyzed five CRC-related genes in patients with CRC [19]. Among these genes, *APC* exhibited the highest frequency of methylation in both mucosa and tumor tissue, yet the methylation level in mucosa remained below 10 % and did not correlate with that in tumors. Conversely, Ramirez et al., in a methylation analysis of six genes including *MLH1* and *p16INK4a*, found that up to 71 % of patients exhibited similar methylation patterns between mucosa and tumor tissues [13]. Additionally, 47 % of the mucosa samples displayed methylation in one or two loci, while 12 % showed methylation in three or more loci. The concept of “field cancerization” has been proposed in normal mucosa adjacent to tumor cells [24]. The presence of hypermethylation in mucosa cells of patients with colon cancer indicates that these cells harbor some of the epigenetic alterations associated with the tumor [25]. Such alterations in the mucosa surrounding CRC lesions may serve as predictors of outcome. As demonstrated in a previous study, hypermethylation of *p16INK4a* in CRC mucosa was associated with a worse disease-free survival [26].

The incidence of CRC is progressively rising, particularly in low- and middle-income countries, likely due to shifting demographics and dietary patterns [27]. Notably, Asians relocating to the United States eventually develop a similar CRC incidence as native-born Americans [28], suggesting a potential influence of dietary habits. It has been posited that the prevalent vegetarian diet in Asian and African societies may confer protection against CRC [29]. Obesity, strongly linked to CRC, is also recognized as a risk factor for DNA hypermethylation [30, 31]. In Sweden, the prevalence of obesity has been steadily increasing over recent decades, whereas Nepalese patients may experience undernourishment comparatively [32]. Obesity could thus contribute to a higher incidence of DNA hypermethylation and CRC development in Swedish patients compared to Nepalese patients. Moreover, dietary differences may play another significant role: the predominant Nepali vegetarian diet may shape a gut microbiota distinct from that resulting from a typical Western-style diet prevalent in Sweden. Additionally, environmental factors vary between the two populations. For instance, Kathmandu, the capital of Nepal, faces significant pollution, and its tap water is often contaminated with bacteria and parasites like Giardia Lamblia. These diverse factors, known to influence DNA methylation and subsequent gene regulation and expression, underscore the disparities between the Nepalese and Swedish populations [18].

Currently, we can only speculate on the reasons for the high methylation levels found in the mucosa of Nepalese patients. One hypothesis would be that aberrant methylation may be triggered in large areas of the gut in response to alterations in the microbiota. The interesting connection between the microbiota and the onset of CRC is an emerging area of research that is garnering increasing attention. For example, *Fusobacterium nucleatum* has been linked to promoter methylation of *MLH1* and *p16INK4a*, while *Streptococcus* spp. seem to be associated with *MLH1* hypermethylation [33]. In another recent study involving mice, even a nonpathogenic microbiota was found to significantly influence DNA methylation in the gut, contributing to the development of aberrant methylomes observed in aging and cancer [34].

With the increasing incidence of CRC among individuals under 50 years old, there is a recent recommendation to lower the age for CRC screening to 45 years [35]. However, routine colonoscopy screening may not be feasible in all countries, particularly those with low CRC incidence. Nevertheless, analyzing methylation in mucosa samples obtained during colonoscopy could serve as a screening tool for high-risk patients and potentially as a follow-up tool for predicting recurrent disease. Moreover, ongoing research is focused on developing and investigating novel techniques for detecting and quantifying methylated DNA using less invasive methods.

The carcinoembryonic antigen (CEA) marker is utilized to monitor treatment response in patients with CRC, while the Fecal Occult Blood Test (FOBT) has been employed for screening high-risk populations; however, its sensitivity and specificity, particularly for proximal CRC, are limited [36]. Given this context, detecting epigenetic alterations in body fluids could offer a safer and more effective screening method for CRC in high-risk populations [27]. Recently, the U.S. Food and Drug Administration (FDA) approved methylated Septin 9 (SEPT9) as a diagnostic marker for CRC screening. SEPT9 exhibits higher sensitivity than the fecal immunochemical test (FIT) and CEA in CRC detection [37]. Nevertheless, SEPT9 analysis is blood-based, and utilizing methylation tests based on fecal samples could provide an effective noninvasive alternative for diagnosing, screening, and monitoring CRC. However, CpG island methylation of certain tumor suppressor genes, including *MLH1* and *p16INK4a*, can also be detected in both mucosal and fecal DNA of healthy individuals. Therefore, it is crucial to identify informative CRC-associated markers whose levels significantly differ from the normal baseline [17].

### Strengths and limitations of study

Extensive research on CRC epigenetics has been conducted in Western populations, but little is known about this phenomenon in resource-constrained countries like Nepal. To our knowledge, this study marks the first investigation of tumor suppressor gene methylation in tumor and mucosa tissue among a Nepalese population with CRC. However, the study is subject to limitations, primarily the relatively small number of subjects studied. This limitation stemmed mainly from the low incidence of CRC in Nepal. In developing countries such as Nepal and India, a considerable proportion of patients with CRC exhibit metastasis at diagnosis, and a significant percentage undergo palliative procedures, precluding the collection of tissue from mucosa and tumors [8, 38]. Nevertheless, the study groups were matched for age and sex, which is a strength, given that these covariates are often associated with DNA methylation status.

There was a concern that the results might have been biased due to the use of different tissue preservation methods in the two patient cohorts. However, upon comparing methylation data from matched FFPE and fresh-frozen tumors, a high degree of similarity was observed. Moreover, Oliveira et al. [39] investigated the performance of DNA methylation analysis in FFPE vs. fresh-frozen ovarian cancer samples and found overall DNA methylation profiles to exhibit high concordance. Additionally, Moran et al. [40] generated single-base resolution DNA methylation profiles covering approximately 5.5 million CpG sites across the genome. Profiles from matched FFPE and fresh-frozen samples were compared to assess the impact of FFPE-related artifacts on methylation calls, and no significant inadequacies were identified. *p16INK4a* methylation status was unavailable for eight mucosa and tumor samples from the Nepalese patient cohort. The negative pyrosequencing results may have been due to formalin-induced DNA degradation, affecting bisulfite conversion and PCR amplification of the DNA. However, DNA of sufficient quality could be extracted from the remaining FFPE samples, as indicated by the internal control assessing successful bisulfite treatment and the high CpG peaks in the pyrograms.

## Conclusions

Nepalese and Swedish patients displayed equal levels of *MLH1* and *p16INK4a* methylation in tumors, but Nepalese patients had a higher level of *MLH1* and *p16INK4a* methylation in mucosa compared to Swedish patients. These discrepancies may be attributed to environmental and lifestyle factors known to influence gene regulation through epigenetic mechanisms, including DNA methylation. Whether the elevated hypermethylation level in the mucosa of Nepalese patients with CRC represents an early aberration during tumorigenesis that could be valuable for screening high-risk patients or predicting recurrence is currently unknown. Ongoing studies will address this question by assessing the prevalence of tumor suppressor gene hypermethylation in the colorectal mucosa of Nepalese control subjects.

## Supplementary Material

Supplementary Material
