# Supplementary material for: Epigenetic differences in the tumor suppressor genes MLH1 and p16INK4a between Nepalese and Swedish patients with colorectal cancer
Source: Innov Surg Sci. 2024 Jul 3;9(3):153–63. doi: 10.1515/iss-2023-0039 (PMC11416036; doi:10.1515/iss-2023-0039)
Supplement: Supplementary file 1 — Supplementary Material [file j_iss-2023-0039_suppl_001.pdf]

Epigenetic differences in the tumor suppressor genes *MLH1* and *p16INK4a* between Nepalese and Swedish patients with colorectal cancer

Bikal Ghimire, Göran Kurlberg, Peter Falk, Yogendra Singh and Yvonne Wettergren

Supplementary material

Supplementary Table 1. Comparison of methylation levels in matching FFPE and fresh-frozen tumor tissue.

Assay	Case	Methylation (%) at respective CpG site	
		FFPE	Fresh-frozen
MLH1	1	13,10,5,7,8	18,13,6,10,10
MLH1	2	3,2,2,3,2	4,1,1,1,1
MGMT[a]	3	88,86,100,86,100	80,73,91,82,86
p16INK4a	4	40,41,39,40,35	63,51,52,56,48
LINE-1[b]	5	67,61,69	61,64,67

[a]PyroMark Q24 CpG MGMT methylation detection assay, Cat. No. 970032
[b]PyroMark Q24 CpG LINE-1 methylation detection assay, Cat. No. 970042

Supplementary Table 2. Number of cases with *MLH1* hypermethylation at respective CpG site in tumor and mucosa.

	Nepalese patients (n = 39)		Swedish patients (n = 39)	
	No. of cases	(%)	No. of cases	(%)
Mucosa				
CpG site 1	12	30.8	1	2.6
CpG site2	12	30.8	1	2.6
CpG site3	11	28.2	1	2.6
CpG site4	11	28.2	1	2.6
CpG site 5	12	30.8	1	2.6
Tumor				
CpG site 1	5	12.8	8	20.5
CpG site 2	6	15.4	6	15.4
CpG site 3	4	10.3	6	15.4
CpG site 4	4	10.3	6	15.4
CpG site 5	5	12.8	7	18.0

Supplementary Table 3. Percentage of patients with *MLH1* hypermethylation at respective CpG site by age group.

Percentage of patients with *MLH1* hypermethylation in mucosa

	CpG site 1		*CpG site 2*		*CpG site 3*		*CpG site 4*		*CpG site 5*	
**Age group**[a]	Nepalese	Swedish	Nepalese	Swedish	Nepalese	Swedish	Nepalese	Swedish	Nepalese	Swedish
<50 years	25.0	9.1	25.0	9.1	16.7	9.1	25.0	9.1	25.0	9.1
50-60 years	33.3	0	33.3	0	33.3	0	20.0	0	26.7	0
>60 years	41.7	0	41.7	0	41.7	0	50.0	0	50.0	0

Percentage of patients with *MLH1* hypermethylation in tumor

	CpG site 1		*CpG site 2*		*CpG site 3*		*CpG site 4*		*CpG site 5*	
**Age group**[a]	Nepalese	Swedish	Nepalese	Swedish	Nepalese	Swedish	Nepalese	Swedish	Nepalese	Swedish
<50 years	8.3	18.2	8.3	9.1	8.3	9.1	8.3	9.1	8.3	9.1
50-60 years	20.0	12.5	26.7	6.2	13.3	6.2	13.3	6.2	20.0	12.5
>60 years	25.0	33.3	25.0	33.3	8.3	33.3	16.7	33.3	8.3	33.3

[a]The number of Nepalese patients <50 years, 50 – 60 years, and >60 years of age were 12, 15, and 12, respectively, whereas the number of Swedish patients were 11, 16, and 12, respectively.

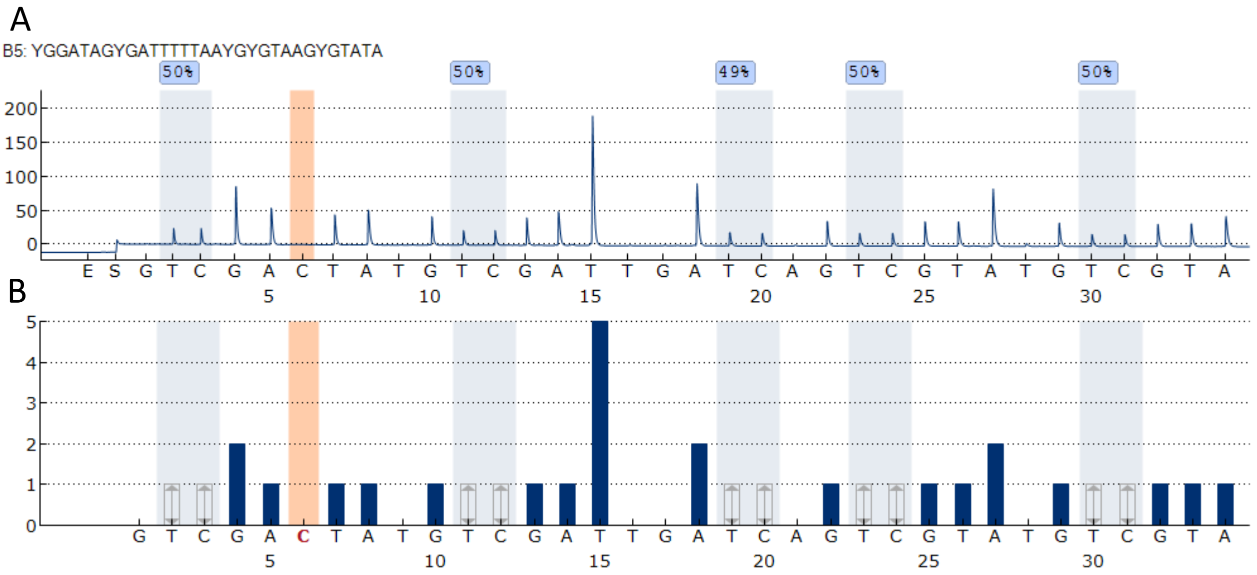

Supplementary Figure 1. A) Pyrogram showing percent methylation at each analysed *MLH1* CpG site in a mucosa sample of a Nepalese patient, and B) Histogram for the *MLH1* assay. Controls for completion of bisulfite treatment are highlighted in orange.

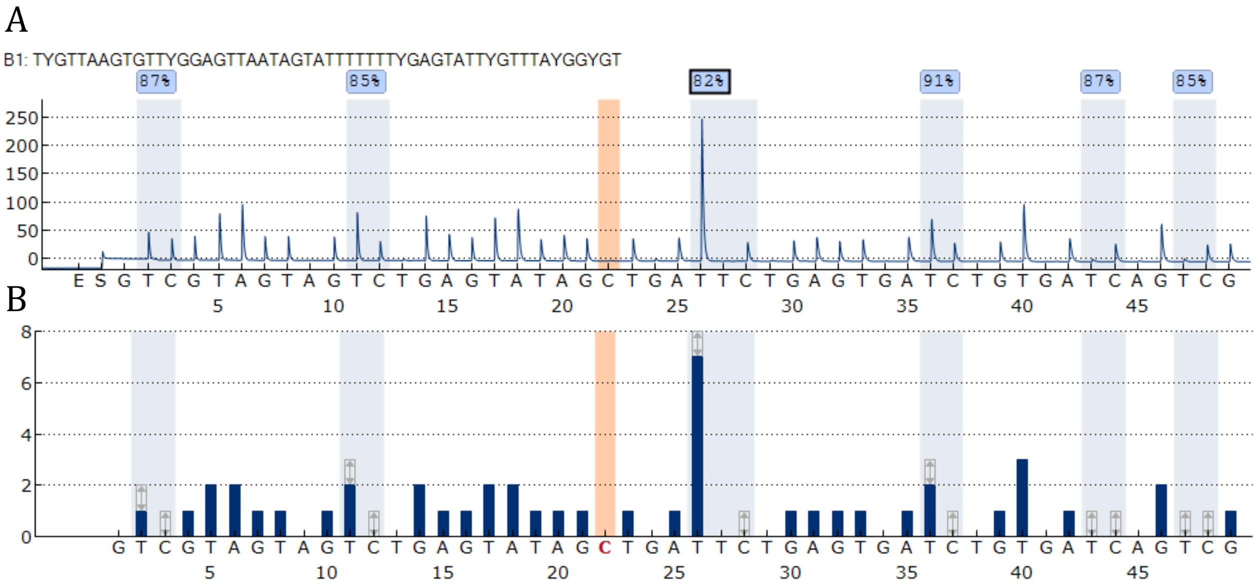

Supplementary Figure 2. A) Pyrogram showing percent methylation at each analysed *p16INK4a* CpG site in a tumor sample of a Nepalese patient, and B) Histogram for the *p16INK4a* assay. Controls for completion of bisulfite treatment are highlighted in orange.

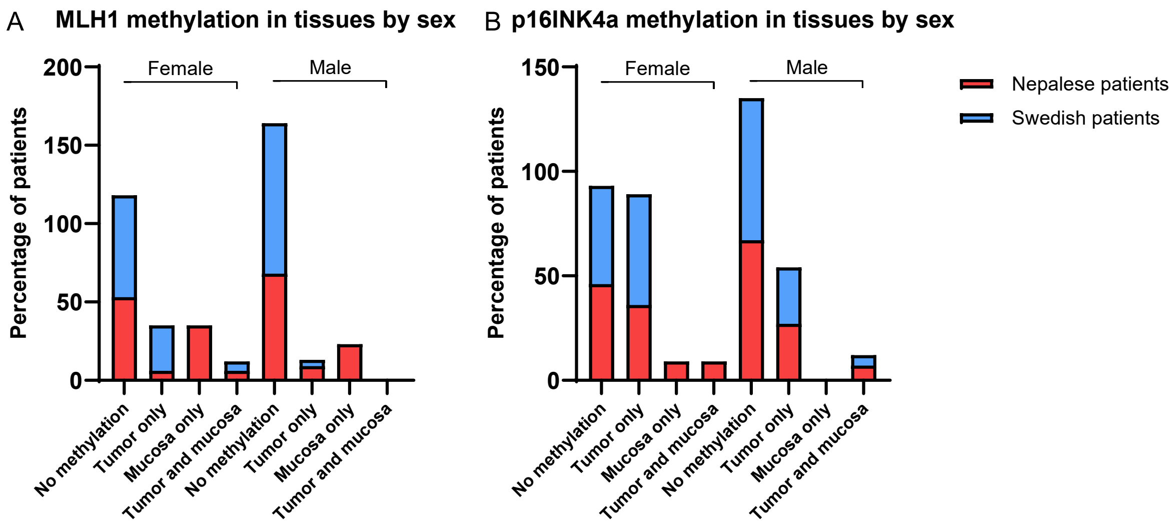

Supplementary Figure 3. Bars and boxes comparing A) the *MLH1* and B) the *p16INK4a* methylation status in tissues deriving from Nepalese and Swedish patients with colorectal cancer by sex. The methylation status is classified as follows: no methylation, methylation only in tumor tissue, methylation only in mucosal tissue, or methylation in both tumor and mucosa.
